# Robotic vs open Puestow procedure for chronic pancreatitis: an updated 15-year experience

**DOI:** 10.1007/s00464-026-12914-2

**Published:** 2026-06-05

**Authors:** Marleny N. F. Araujo, Valeria Arango, Raphael L. C. Araujo, Erin Baker, Dionisios Vrochides, John B. Martinie

**Affiliations:** Division of HPB Surgery, Department of Surgery, Carolinas Medical Center, 1025 Morehead Medical Drive, Suite 600, Charlotte, NC USA

**Keywords:** Puestow, Chronic pancreatitis, Pancreaticojejunostomy, Robotic surgery

## Abstract

**Introduction:**

Pain from chronic pancreatitis (CP) impairs quality of life and demands continuous treatment, whether pharmacological, endoscopic or surgical. Surgical treatment for CP can include solely decompression, resection, or a combination of both. The modified Puestow [1] procedure is an established indication for patients with a dilated pancreatic duct and without the need for parenchyma resection.

**Methods:**

We report a series of 48 patients who have undergone a Puestow procedure from January 2009 to July 2025 (28 open and 21 robotic).

**Results:**

This is a retrospective review of a prospectively maintained database. Comparison between groups reveals a shorter overall hospital length of stay and zero readmissions among the robotic group (respectively *p* = 0.036 and 0.029), as well as no difference in morbidity or operative time when compared to open cases.

**Conclusion:**

At our high-volume HPB center, robotic Puestow procedures are safe and feasible, with similar complication rates and earlier discharge.

**Supplementary Information:**

The online version contains supplementary material available at 10.1007/s00464-026-12914-2.

Management strategies for pain due to chronic pancreatitis (CP) range from pharmacological, which often fail to provide long-term pain relief and might lead to opioid dependence and resistance, to other interventions such as celiac plexus blocks and ERCP (endoscopic retrograde cholangiopancreatography), which are associated with higher reintervention rates compared to surgical options [1]. Chronic pain significantly impairs quality of life, and surgical decompression of the pancreatic duct has proven to have favorable long-term outcomes with partial or complete pain relief [2–4]. Nevertheless, the ideal timing to perform surgery remains controversial, as it is unclear whether there is greater benefit to operating earlier or later in the course of the disease.

Surgical treatment offers an alternative, with techniques broadly categorized into resection and drainage procedures or a mixture of both. The modified Puestow lateral pancreaticojejunostomy (LPJ) procedure was published by Partington and Rochelle, in which they preserved the tail of the pancreas and extended the opening of the pancreatic duct, without the need for resection and splenectomy [5]. This procedure has demonstrated, among other pancreatic surgeries (Frey, Whipple), durable pain relief, low complication rates, and favorable postoperative outcomes in the management of chronic pancreatitis [1]. Recent advances in robotic surgery have further reduced postoperative complications, making the robotic Puestow procedure a safe and feasible minimally invasive option for drainage in chronic pancreatitis [6–9].

The purpose of this study is to update our previously published case series of robotic cases (2009–2015) by presenting an expanded 10-year experience while comparing the outcomes of open and robotic approaches.

## Methods

We conducted a retrospective analysis of our prospectively maintained database including all patients who have undergone longitudinal pancreaticojejunal anastomosis without associated pancreatic resection (Puestow procedure), from January 2009 to July 2025.

The robotic pancreatic surgeries were performed exclusively by two surgeons in our HPB department. The selection criteria for this approach included patients without a contraindication for pneumoperitoneum, no more than 3 previous abdominal surgeries, and no complex ventral hernias.

Descriptive analysis was reported as a median and range for continuous variables and total number and percentages for categorical variables. We analyzed demographic data, intraoperative results, and postoperative outcomes. For comparative statistical analysis, we used a nonparametric Mann–Whitney test for continuous variables, Fisher’s exact test for categorical data, and ANOVA for variables with 3 or more groups. Significance was considered if *p* < 0.05. For statistical analysis, SPSS® software (Microsoft) was used.

### Operative technique

Open LPJ is performed by all HPB surgeons via midline laparotomy extending from the xiphoid process to the umbilicus. The round ligament is isolated and preserved for use as a vascularized pedicle flap. The stomach is elevated, and the retroperitoneum is entered via the gastrocolic ligament. The gastrocolic ligament is divided with use of advanced energy devices. The stomach is retracted cephalad and caudally, respectively, to expose the pancreas. Inflammatory adhesions to the posterior surface of the stomach are mobilized as needed to facilitate exposure. The location of the main pancreatic duct is identified using intraoperative ultrasound (BK Ultrasound, Herlev, Denmark), and the duct is aspirated with a small-caliber needle to confirm the presence of clear pancreatic fluid. Stones within the pancreatic head and duct are also identified via intraoperative ultrasound. A pancreatic ductotomy is created with electrocautery and extended proximally and distally to within 0.5 cm of the duodenal wall. Reconstruction is performed by identifying a loop of jejunum 40 cm distal to the ligament of Treitz and dividing it with a stapling device. The distal end of the transected bowel is brought through the avascular space of the transverse colon mesentery and apposed to the pancreatic ductotomy. The antimesenteric border of the bowel is opened with cautery. A two-layered pancreaticojejunostomy was performed early in the series with an inner layer of interrupted duct-to-mucosa 3-0 Prolene® (polypropylene; Ethicon, Somerville, NJ, USA) suture and an outer layer of interrupted 3-0 silk suture. Since 2013 open LPJ has been created using a running 4-0 V-Loc™ suture (polydioxanone; Covidien/Medtronic, Minneapolis, MN, USA); 4 VLoc sutures are typically required to complete the anastomosis. A stapled jejunojejunostomy is created to restore gastrointestinal continuity. The preserved round ligament is then wrapped around the pancreaticojejunostomy. An aerosolized fibrin glue (Tisseel™, Baxter Healthcare Corporation, Deerfield, IL, USA) is used to reinforce the anastomoses, and a closed suction drain (C. R. Bard, Inc., Covington, GA, USA) is placed adjacent to the pancreaticojejunostomy prior to abdominal closure.

**Fig. 1:**
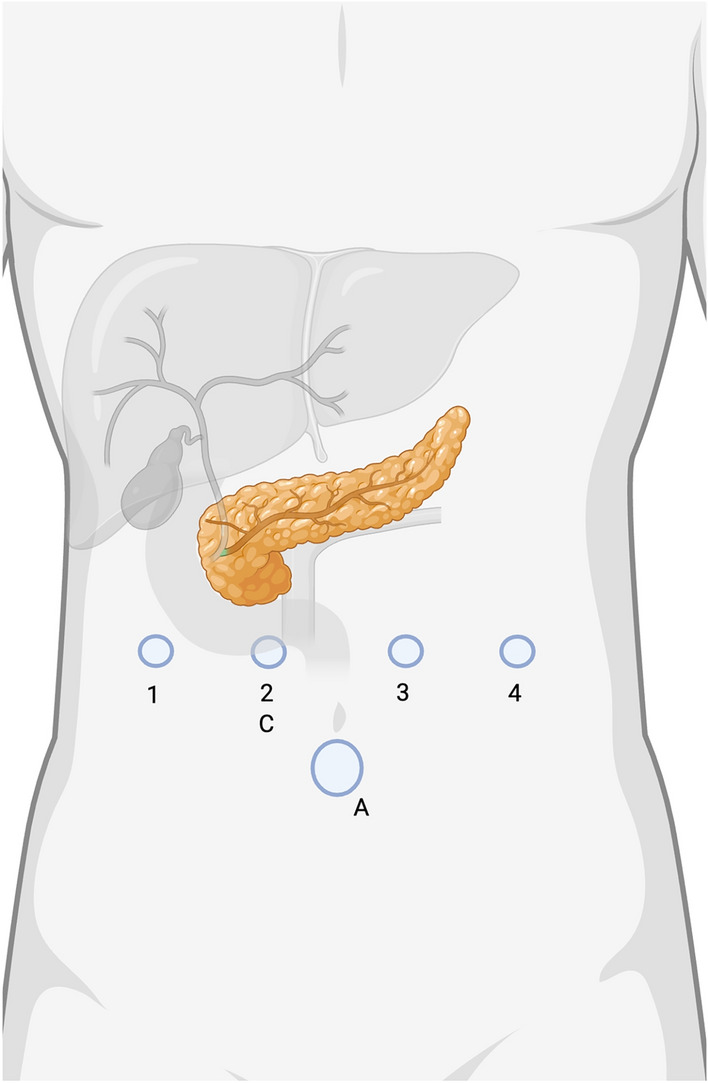
Trocar placement (Created in BioRender. Araujo, R. https://BioRender.com/1nyc4rk)

**Fig. 2:**
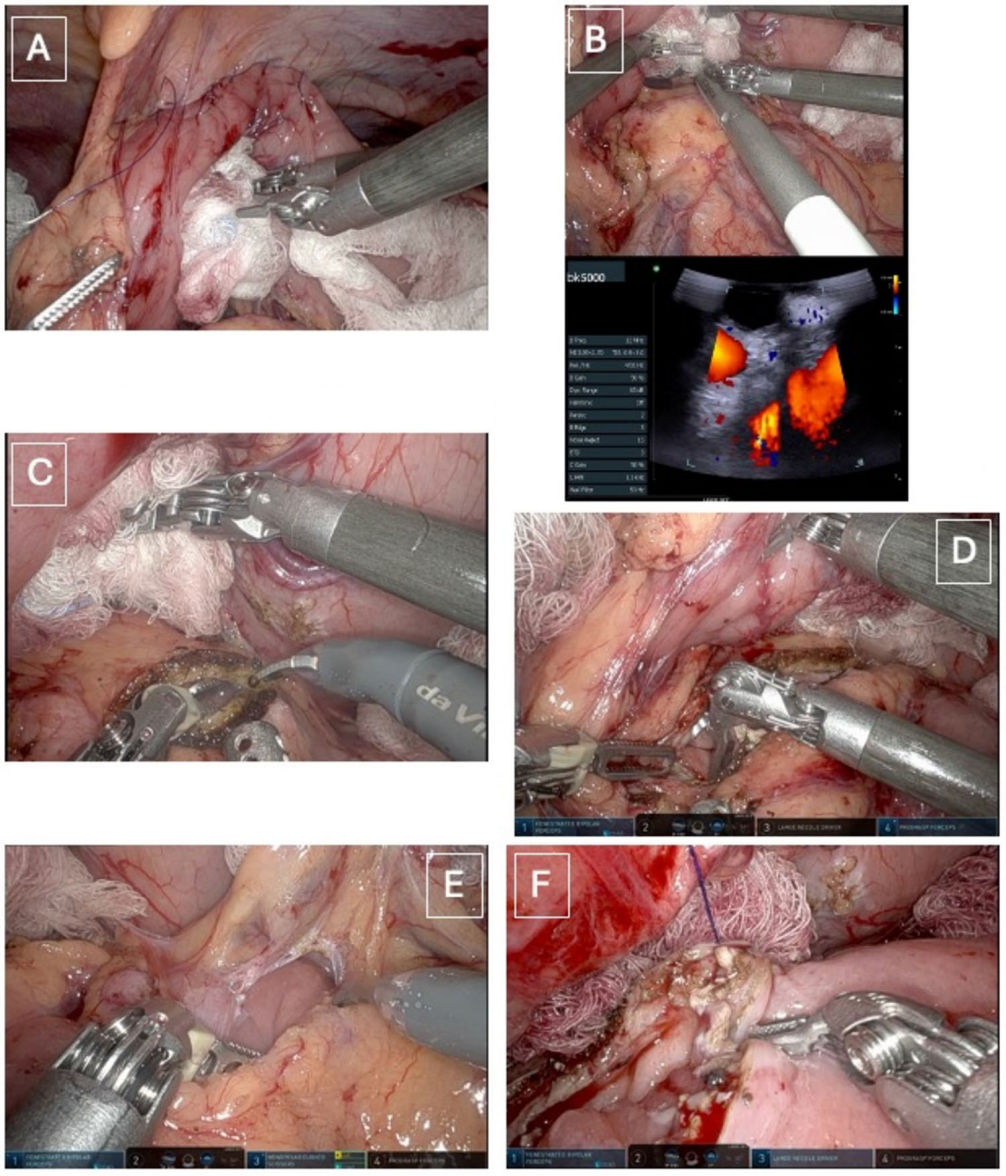
Robotic steps. **A** stomach is sutured to the anterior abdominal wall for better exposure; **B** ultrasound to locate the pancreatic duct; **C** ductotomy using monopolar energy; **D** exploration of pancreatic duct after ductotomy to remove stones; **E** opening of transverse mesocolon to create a window for the jejunum to reach the supramesocolic area for the pancreaticojejunal anastomosis; **F** creation of the pancreaticojejunal anastomosis with VLoc running sutures

**Fig. 3:**
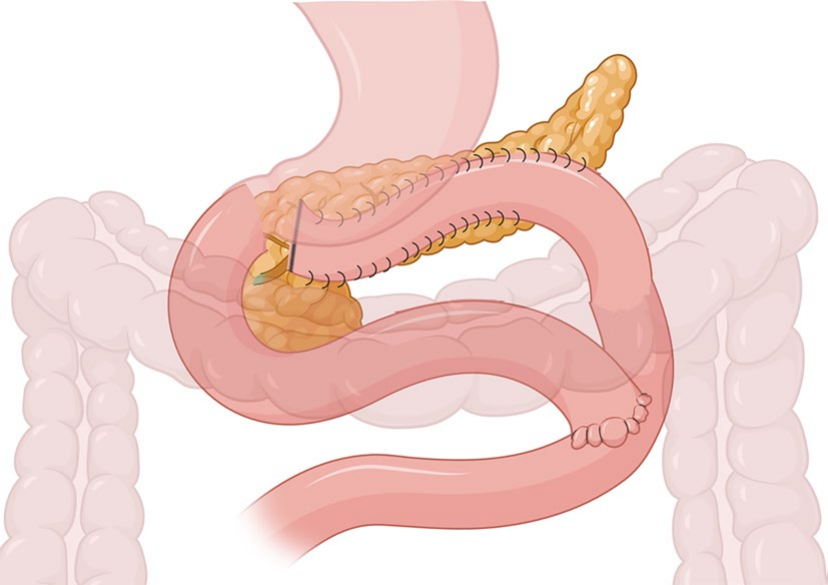
Illustration of the final aspect of anastomoses

**Fig. 4:**
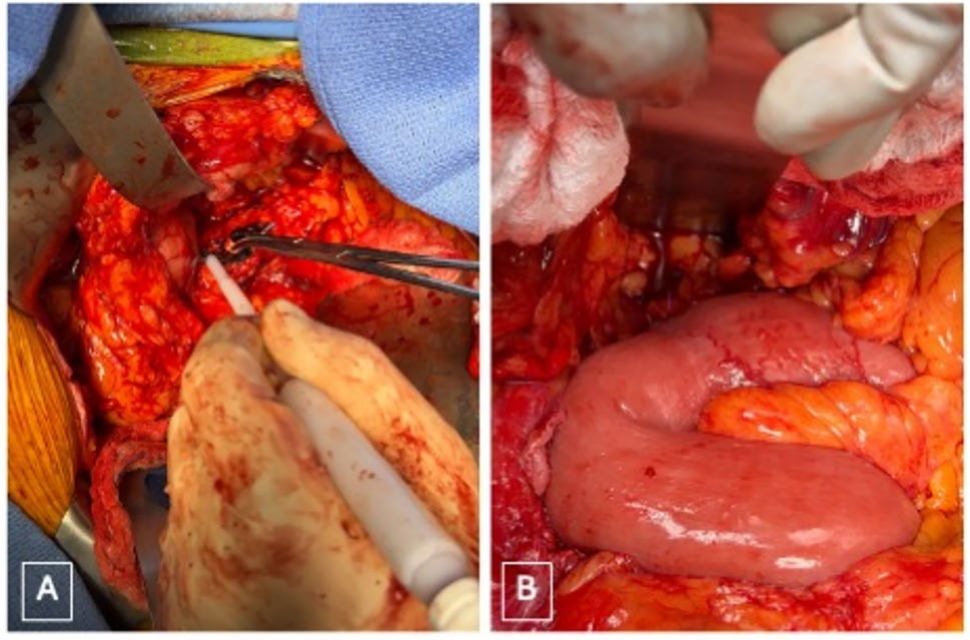
Open procedure. **A** Exploration of pancreatic duct with removal of stones; **B** final aspect of lateral pancreatojejunostomy

With advances in the da Vinci® robot (Intuitive Surgical, Sunnyvale, CA, USA), currently we do the port placement as shown in Fig. 1, with 4 trocars in a horizontal line about 2 cm above the umbilicus line and an auxiliary trocar in the suprapubic area. The camera is located on the left side of the midline. The patient is placed in the supine position and pneumoinsufflation is obtained with a Veress needle at the upper left quadrant. After abdominal entry, the round ligament is taken down from the posterior surface of the abdominal wall and preserved for a pedicle flap as in open LPJ. Entry into the retroperitoneum is performed with the vessel sealer device, and the stomach is sutured to the abdominal wall with running V‐Loc suture to facilitate exposure. Intraoperative ultrasound is used to confirm the position of the main pancreatic duct and any ductal stones. The main pancreatic duct is entered using the monopolar shears until clear pancreatic fluid returns. Curved dissectors are inserted into the main pancreatic duct to elevate the tissue prior to division with monopolar energy. The ductotomy is continued to within 0.5 cm of the duodenal wall, and the duct and pancreatic head are explored to remove any ductal stones. The jejunum is transected 30–40 cm distal to the ligament of Treitz with a robotic stapler. The distal end of the transected bowel is brought into the retroperitoneum in a retrocolic fashion, and a jejunostomy is created along the antimesenteric border of the bowel to approximate the length of the pancreatic ductotomy. A duct-to-mucosa longitudinal pancreaticojejunostomy is created in running fashion using 4-0 V‐Loc suture. Typically, four sutures are required to complete the anastomosis. A stapled jejunojejunostomy is created with the robotic stapler. The pancreaticojejunostomy is reinforced with the round ligament and sprayed with aerosolized fibrin glue. A closed suction drain is placed adjacent to the pancreaticojejunostomy and the abdomen is then deflated. The port sites are closed with polydioxanone suture in interrupted fashion (see Figs. 2, 3, 4).

## Results

From our database, we identified 48 consecutive patients who underwent Puestow procedures in a 16-year period; 27 underwent open procedures, and 21 robotic procedures. All patients had a diagnosis of chronic pancreatitis, and chronic pain was the indication for surgery in all cases.

Overall, the median age was 52.5 years (range 12–84) and 28 (58.3%) of patients were male. Sex was the only demographic variable analyzed with a significant difference between groups (the robotic group 38% male and the open group 74% male; *p* = 0.019), as demonstrated in Table 1. Regarding perioperative outcomes, 3 robotic surgeries were converted to open, due to technical difficulties (middle colic vessels and stomach fused to anterior surface of pancreas, not being able to identify pancreatic duct and lack of qualified assistant in the first robotic case done). No difference between groups was found regarding blood loss, and 3 patients in the open group received red blood cell transfusion, while none in the robotic group (*p* = 0.246). Median operative time was only 20 min longer in the robotic group than the open group, without statistical significance, as reported in Table 2.

**Table 1:** Demographic characteristics of 48 patients submitted to open or robotic Puestow

Characteristics	Total (*N* = 48)	Robotis (*n* = 21)	Open (*n* = 27)	*p* value
Age	52.5 (12–84)	51 (15–84)	54 (12–74)	0.148
Sex				0.019
Male	28 (58.3)	8 (38.1)	20 (74.1)	
Female	20 (41.7)	13 (61.9)	7 (25.9)	
BMI	23.19 (16.6–35.8)	23.09 (16.57–35.34)	23.3 (18.95–35.8)	0.582
ASA score*				0.441
2	8 (16.7)	4 (19)	2 (7.4)	
3	34 (70.8)	16 (76.2)	20 (74.1)	
4	2 (4.2)	1 (4.8)	1 (3.7)	

*4 missing values on open group (ASA score)

**Table 2:** Intraoperative data of 48 patients submitted to open or robotic Puestow

Characteristics	Total (*N* = 48)	Robotic (*n* = 21)	Open (*n* = 27)	*P* value
Conversion		3 (14%)		
EBL (mL)	100 (20–600)	75 (25–450)	100 (20–600)	0.165
Intra-op transfusion (RBC)	3 (6.3)	0	3 (11.1)	0.246
Operative time (min)	231 (131–554)	249 (157–554)	228 (131–423)	0.174

Comparing short-term surgical outcomes, hospital length of stay was 2 days longer in the open group (7 vs 5 days; *p* = 0.036), and 3 subjects (22.2%) in the open group were readmitted within 30 days of surgery, while none in the robotic group (p = 0.029). The overall complication rate was 60%, with the majority being Clavien–Dindo 1 and only 20% severe complications (Clavien-Dindo > 3). No deaths occurred (see Table 3).

**Table 3:** Postoperative outcomes of 48 patients submitted to open or robotic Puestow

Characteristics	Total (*N* = 48)	Robotis (*n* = 21)	Open (*n* = 27)	*p* value
Clavien				0.88
0	18 (37.5)	5 (23.8)	13 (48.1)	
I	17 (35.4)	11 (52.4)	6 (22.2)	
II	3 (6.3)	2 (9.5)	1 (3.7)	
IIIa	4 (8.3)	0	4 (14.8)	
IIIb	5 (10.4)	3 (14.3)	2 (7.4)	
IVa	1 (2.1)	0	1 (3.7)	
Clavien > III	10 (20.8)	3 (14.3)	7 (25.9)	0.478
Pancreatic fistula	4 (8.4)	2 (9.5)	2 (7.4)	0.594
Reoperation	2 (4.2)	0	2 (7.4)	0.497
Length of hospital stay	6 (2–25)	5 (2–25)	7 (3–24)	0.036
Readmission (30 days)	6 (12.5)	0	6 (22.2)	0.029

## Discussion

Our study demonstrates a series of patients from a single institution submitted to the robotic Puestow procedure for the treatment of chronic pancreatitis, reinforcing its feasibility and safety. In the robotic group, 15% of patients had major complications (2 fistulas without the need for reintervention) compared to 26% in the open group. Although this did not reach statistical significance, length of hospital stay and readmission rates were better in the robotic group. These results reinforce previous results from our group where we compared 19 open and 7 robotic Puestows from 2009 to 2015 [8]. At that time, adoption of robotics for pancreatic surgery was rising and cost of care was under debate, and one study from our group already demonstrated that median operative supply cost was higher in the robotic group, but median total cost was lower for the robotic group, possibly linked to fewer complications and shorter hospital stay [8]. Even though we did not perform cost analysis in our study, we still found similar results regarding those two operative outcomes, as well as no reoperations or readmissions in the robotic group.

As far as we know, this is the largest number of robotic Puestow procedures reported so far. The first case series in this technique was reported by Fernandes & Giulianotti in 2013 [3], who published results of 8 patients submitted to Puestow, along with other robotic procedures regarding pancreatic surgery. Their group subsequently reported 6 more patients in a new study, where they also described their technique for the robotic Puestow [6]. Whereas the first study shows a resolution of pain symptoms in 80% of patients, after those 14 cases they update their results, reporting an overall rate of symptom resolution of 71%. Hamad et al. published results from their group with 8 robotic Puestow, with median operative time of 210 min and median EBL of 45 ml, without need for intraoperative blood transfusions, comparable to ours. They reported median LOS of 7 days and one grade A fistula [9].

Pain is the main indication for surgery in chronic pancreatitis and surgery has the potential of complete resolution of symptoms or at least partial resolution, leading to less need for pain medication, something very important since many patients use opioids chronically and not only dependence is an issue, but the effect of the medication waives over time. Pharmacological control of pain is the first line of treatment, and decompression of the pancreatic duct via endoscopy (ERCP) is usually attempted before surgery becomes an option. Celiac plexus block was indicated for patients with CP, but pain relief is maintained in a short number of patients after 6 months [1, 10] and it seems currently to be more suitable for patients with unresectable malignancy.

Some retrospective multicenter series have demonstrated that some patients can achieve significant pain relief with endoscopic therapy only (median of 3 ERCPs), meaning that 30% of patients might achieve full resolution and do not need surgery [11, 12]. Two randomized controlled trials have shown the superiority of surgery for long-term resolution of symptoms when compared to endoscopic procedures. Dite et al. [13] reported satisfactory short-term results regarding pain relief with both interventions, but a high rate of non-responders in the ERCP treatment arm after 5-year follow-up (35%) was observed compared to 14% in the surgery arm. Similar results were found by Cahen et al. [14] in 2011, with 32% of patients achieving partial or complete pain resolution in the endoscopy group, while 75% showed at least partial relief after surgical treatment, with results maintained after a long follow-up of 7 years.

Usually there is substantial pain relief after surgical procedures for CP, up to 80–100%, and more recently, Van Veldhuisen et al. [15] have reported a nationwide consensus from the Netherlands with 83% of patients submitted to the Puestow procedure achieving pain relief and only 17% of patients still using opioids after a median follow-up of 11 months. Different surgical techniques can be indicated for the treatment of chronic pancreatitis, and they should be tailored to patient anatomy and symptoms. According to a recent international consensus, Puestow and Frey procedures seem to be suitable for patients with a dilated duct and a normal-size pancreatic head, with equivalent pain control [16].

Our study has limitations inherent to observational and retrospective studies. Concerning clinical outcomes, the primary goal of surgical treatment for chronic pancreatitis is pain relief; however, because most patients did not follow up with our department, we were unable to obtain a reliable measure of this outcome from either approach.

The number of open procedures was higher than that of robotic ones, as a reflection of the transition of surgical approaches from open to robotic approaches over the years. Nevertheless, neither differences in the robotic learning curve nor surgeon discernment for surgical approach could be excluded since not all surgeons of the department had migrated to the robotic approach as their main surgical preference.

Robotic Puestow procedure can be challenging due to inflammation of the pancreas, the need of exploration of the pancreatic duct with extraction of stones, and potential bleeding, contributing to high morbidity rates. Despite that, in our study, the median EBL in the robotic group was 75 mL and 100 mL in the open group. Operative time was not considerably higher in the robotic group, and we associate this with the extensive expertise in robotic HPB surgeries that our group has, with more than 2000 cases (liver and pancreas).

This series comparing robotic and open Puestows is an update from a previous series from our group [8] that has focused on the cost of robotic surgery compared to open Puestows, as well as overall outcomes. The conversion rate dropped from 22 to 14%, with only 1 conversion for the past 10 years (12 cases). It points to a learning curve, as in all pancreatic surgeries performed robotically. We believe that robotic Puestow is a good starting point in the learning curve of surgeons that wish to gain more experience with pancreatic surgery, since resection or vascular dissection is not necessary.

## Conclusion

The modified Puestow procedure is a feasible and safe choice for the treatment of patients with chronic pain due to CP, and the robotic platform seems to be associated with slightly lower morbidity rates and can contribute to earlier discharge from the hospital.

## Supplementary Information

Below is the link to the electronic supplementary material.

Supplementary file1 (DOCX 16 kb)
