# Supplementary material for: Robotic vs open Puestow procedure for chronic pancreatitis: an updated 15-year experience
Source: Surg Endosc. 2026 Jun 5;40(7):6323–9. doi: 10.1007/s00464-026-12914-2 (PMC13369739; doi:10.1007/s00464-026-12914-2)
Supplement: Supplementary file 1 — Supplementary file1 (DOCX 16 kb) [file 464_2026_12914_MOESM1_ESM.docx]

Supplementary material

Distribution of cases of Puestow procedures performed per year, according to approach (open or robotic). Some gaps are found due to change in eletronic medical record software.

| Year | Open | Robotic |
| --- | --- | --- |
| 2008 | 1 |  |
| 2009 | 2 | 2 |
| 2010 | 2 |  |
| 2011 | 1 |  |
| 2012 |  | 1 |
| 2013 | 5 | 1 |
| 2014 | 4 | 1 |
| 2015 | 4 | 3 |
| 2016 |  | 2 |
| 2017 |  | 3 |
| 2018 | 1 | 2 |
| 2019 |  | 1 |
| 2020 |  | 2 |
| 2021 |  | 1 |
| 2022 |  | 1 |
| 2023 | 4 |  |
| 2024 | 1 | 1 |
| 2025 | 2 |  |
